# Evaluating the impact of the ICNET® clinical decision support system for antimicrobial stewardship

**DOI:** 10.1186/s13756-019-0496-4

**Published:** 2019-03-06

**Authors:** Katie L. Heard, Stephen Hughes, Nabeela Mughal, Berge S. Azadian, Luke S. P. Moore

**Affiliations:** 1 Chelsea and Westminster NHS Foundation Trust, 369 Fulham Road, London, SW10 9NH UK; 2 North West London Pathology, Fulham Palace Road, London, W6 8RF UK; 3 National Institute for Health Research Health Protection Research Unit in Healthcare Associated Infections and Antimicrobial Resistance, Imperial College London, Hammersmith Campus, Du Cane Road, London, W12 0NN UK

**Keywords:** Antimicrobial resistance, Antimicrobial stewardship, Clinical decision support system, eHealth, mHealth

## Abstract

**Background:**

Antimicrobial resistance (AMR) is an ecological and economic crisis and stewardship of available antimicrobials is required. Electronic prescribing, where available, enables auditing of practice, yet in order to be efficient and effective in addressing inappropriate antimicrobial prescribing, better use of current and new technological interventions is needed. This retrospective observational evaluation looked at the impact of a commercial clinical decision support system (CDSS) on the workflow of an established antimicrobial stewardship (AMS) team.

**Material/methods:**

Clinical, workflow, and pharmaceutical data from 3 months post implementation of CDSS were collated, and compared to the same 3 month periods in preceding years. The evaluation considered total interventions made, the types of intervention made, impact of said interventions, and time spent executing interventions. All antimicrobial data were adjusted for total daily defined doses (DDD) of intravenous antimicrobials.

**Results:**

*Productivity:* In the 3 month evaluation period (Jun-Aug 2016) a total of 264 case reviews resulting in 298 AMS interventions were made. Compared to preceding years where 138 and 169 interventions were made (2013 and 2014 respectively). In 2013 49% of interventions were stopping medication and 30% change of therapy based on cultures and sensitivities compared to 25 and 17% in 2016. In contrast to previous years’, the CDSS instead enabled a greater number of dose/drug optimisation (13%), escalation of antimicrobials (12%) and intravenous (IV) to oral switch (11%) interventions.

*Patient Identification:* Despite increased patient numbers post-CDSS, on average 46 min per day was spent compiling a patient list for review, compared to 59 min in 2014. The use of CDSS facilitated 15 interventions/1000DDD, compared to pre-intervention (9.4/1000DDD in 2013; 11.5/1000DDD in 2014).

**Conclusions:**

Initial evaluation of the impact of this CDSS on AMS at the organisation has demonstrated effectiveness in terms of case finding, AMS team productivity, and workflow auditing. More importantly, patient infection management has been optimised with a shift in the emphasis of AMS interventions. It has contributed to the success of the healthcare provider achieving nationally set remunerated AMS targets.

## Background

Antimicrobial resistance (AMR) is a global threat to health and healthcare provision at [1] [2]. Attempts to address this have included a number of national and international programmes to promote antimicrobial stewardship (AMS) and raise awareness [3]. In the UK an increase in antimicrobial prescribing was observed between 2010 and 2013; a 4% rise in general practice and a 12% increase in hospital inpatient prescribing [4]. To address this UK national strategies have been in place since 2013, with various interventions to raise awareness [5–7]. One of these has been the English Commissioning for Quality and Innovation (CQUIN) targeting AMR, first released in 2016; it comprised 4 targets focussing on AMS (Table 1.), for which there were financial incentives [8]. This intervention has stimulated policy changes around antimicrobial prescribing in inpatient settings in many areas of England, yet difficulties in implementation for AMS teams are widespread.

**Table 1:** NHS England: Commissioning for Quality and Innovation (CQUIN) Indicators and Targets for Antimicrobial Resistance and Antimicrobial Stewardship 2016/2017 [7]

Reduction in antibiotic consumption (part 4a)	
• Reduction of 1% or more in total antibiotic consumption against the baseline	
• Reduction of 1% or more in carbapenem against the baseline	
• Reduction of 1% or more in piperacillin-tazobactam against the baseline	
Empiric review of antibiotic prescriptions (part 4b)	
• Percentage of antibiotic prescriptions reviewed within 72 h	
Quarter 1	Perform an empiric review for at least 25% of cases in the sample
Quarter 2	Perform an empiric review for at least 50% of cases in the sample
Quarter 3	Perform an empiric review for at least 75% of cases in the sample
Quarter 4	Perform an empiric review for at least 90% of cases in the sample

The activity of AMS teams is varied and often limited due time and staffing constraints [9], but includes both strategic (education and training, writing antimicrobial guidelines, policy and horizon scanning), and operational roles, the latter including identifying individual patients for clinical review [10]. Identifying patients suitable for these clinical reviews can be time consuming, leading to AMS teams focusing on high cost, broad spectrum, or ‘restricted’ antimicrobials. Whilst these prospective audit and feedback approaches have been demonstrated to be an effective and integral component of AMS programmes, the need to focus on only certain antimicrobials means opportunities to improve patient outcome, reduce broad spectrum exposure and *C.difficile* rates are often missed [11]. AMS team interventions to improve antimicrobial prescribing may be restrictive or enabling, and while both are effective at increasing compliance, enablement is preferred [12]. In order to address AMR therefore, efficient, effective, clinical reviews of all suboptimal antimicrobial prescriptions need to be enacted, preferably using an enabling approach. To support this widening cohort of patients for AMS team review, electronic prescribing [12] and more advanced clinical decision support systems (CDSS) may aid efficiency and increase the scope of AMS interventions [13, 14].

A systematic review [13] of CDSS for AMS has noted that many analyses failed to look at their wider healthcare impact. This includes a failure to detail the nature of changes to individual prescriptions made as a result of their use, and benefits for prescribers and healthcare organisations. To explore the impact of a commercial CDSS for AMS, we conducted a retrospective observational evaluation investigating productivity, the method (office based vs. bedside), and types of interventions. Productivity was measured by time taken to identify patients and the number of interventions made by the team.

## Methods

In April 2016 a commercial CDSS was introduced at a single site London teaching hospital with an established, multi-professional AMS team. The hospital has 450 beds, serving general medicine, surgery, obstetrics & gynaecology, paediatrics and neonates, with tertiary referral paediatric surgery, plastic surgery, bariatric surgery and burns. There is an 11 bed intensive care unit (ICU)/high-dependency unit (HDU), a 4 bed burns ICU/HDU, and an 11 bed paediatric HDU.

In the years prior to implementation of the CDSS (i.e. pre April 2016), AMS activities focussed on patient level prescribing data being extracted from the JAC® dispensing system. High cost and extended spectrum antimicrobials (e.g. piperacillin-tazobactam and carbapenems) dispensed in the preceding 24 h were collated and assessed for compliance in line with local guidelines by antimicrobial pharmacists with selectively reported microbiology susceptibility results. All non-compliant prescriptions were discussed in an office based multidisciplinary team meeting with a consultant and registrar in microbiology / infectious diseases where access to full laboratory data was available and prescriptions further reviewed for appropriateness. The patients’ medical teams were then contacted via junior doctors and advice given over the phone where discrepancies were identified. This method had reduced impact for a number of reasons; there was little scope to access other clinical information (notes, observation charts), bedside clinical reviews were rare, most conversations were conducted with foundation year doctors who had limited information and are reluctant to challenge the senior doctors of the patient parent team. Further to this, correlating advice given to actual changes to prescribed antimicrobials was difficult.

The CDSS implemented in April 2016 was a commercial system developed by ICNet® (Baxter®). It composites real time microbiology, biochemistry, haematology results (extracted from the Sunquest® laboratory information management system), currently prescribed drugs and demographic information (extracted from the Lastword® electronic patient record system). The CDSS allows users to code (i) alerts and (ii) reports on predetermined patient biochemistry, microbiology, or drug information. Alerts can be set to the user home screen and will notify the user as real time data triggering the alerts are fed into the CDSS. Alerts set by our hospital site include microbiology sterile site growth, new restricted drug prescriptions, and unprecedented changes in white blood cells or liver function test while on an antimicrobial. Reports can be set to identify patients on antimicrobials or that have microbiology with specified resistance patterns. These can then be run for a set time period or patient group. Reports are used to identify patients for audit or for AMS interventions and examples of reports include; patients on defined medications, patients with specific antimicrobial resistance patterns, or those having been on antimicrobials for an extended period of time. The CDSS allows a work list to be compiled; patients on this list can be graded as to whether a review is required or just ongoing monitoring. An anti-microbial pharmacist reviews all triggered alerts and specific reports and adds patients to the ‘work list’. There was no change in workforce size pre- to post-intervention but the AMS workflow changed to an infection pharmacist and doctor meeting on the wards to review pre-identified patients with full access to patient notes, microbiology (including suppressed susceptibility results), biochemistry and haematology. This allowed decisions to be made at patient bedside and potential contraindications including, patient–antimicrobial or drug–antimicrobial interactions to be identified at the time of review. Bedside reviews also enable the team to have increased clinical confidence in giving advice particularly when de-escalating therapy as a clearer clinical picture can be obtained. This also facilitated the AMS team having face to face discussions with the patients’ clinical team. The CDSS provides a constant live feed of prescribing and microbiology data, thus providing the antimicrobial team data throughout the day and preventing a 24 h delay as per the previous ‘daily list system’. Documentation of AMS team user notes within the CDSS allows patient follow up by any of the AMS team and continuity of patient care.

We analysed data on numbers of cases reviewed using the CDSS (a case review is logged whenever a patient is accessed via the CDSS), the number of interventions made, types of intervention, and time spent executing interventions pre and post implementation of the CDSS to ascertain a measure of added value. System utilisation, clinical impact, and prescribing data from 3 months post implementation (Jun-Aug 2016) of CDSS was retrospectively collated from within the CDSS. The post-intervention data was then compared to 3 months of data collected in corresponding periods in 2013 and in 2014. Parameters investigated included: the number of patients reviewed, total interventions made, types of intervention made and the time burden of the daily AMS operational role. All outcomes were adjusted for total daily defined doses (DDD) of intravenous antimicrobials prescribed in the given time period. Due to a number of changes in AMS staffing (pharmacist and locum medical staff), and the iterative implementation of the CDSS system into existing NHS information technology systems, 2015 data was not utilised in this analysis and instead was used as a washout period between pre- and post-intervention.

This report details a service evaluation of the ICNet® software registered with the Joint Research Compliance Office, Chelsea & Westminster Hospital Campus, Imperial College London (ref: CAPP 1327, July 2015).

## Results

### Productivity

**Fig. 1:**
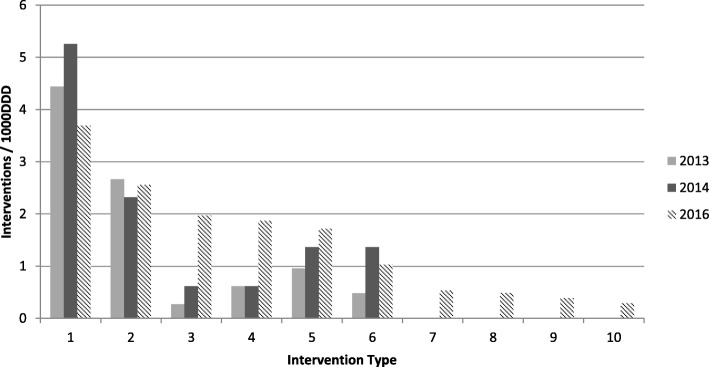
Clinical interventions made pre- (2013 & 2014) and post- (2016) introduction of a commercial computerised decision support system for antimicrobial stewardship (ICNet®). 1. Stop antimicrobial, 2. Change antimicrobial based on microbiology results, 3. Dose / drug optimisation, 4. Escalate antimicrobial therapy in deteriorating patient, 5. Intravenous to oral switch, 6. Stop / change antimicrobial in line with local guidelines, 7. Advise therapeutic drug monitoring / disease monitoring, 8. Start / restart antimicrobials, 9. Multidisciplinary team infectious diseases ward review, 10. Increase duration of antimicrobial therapy

In the 3 month evaluation period the CDSS was used daily (Monday-Friday) for a mean of 2 h 19 min. A total of 2664 case reviews were made on patients over the 3 month period. During the evaluation period 298 clinical interventions where recorded on the CDSS system (Fig. 1). This is in comparison to preceding years where 138 and 169 clinical interventions were made over the same 3 months in 2013 and 2014 respectively. Pre-CDSS interventions were predominantly cessation of antimicrobials, however the trend in interventions changed post implementation of CDSS (Fig. 1). In 2013 49% of interventions were stopping medication compared to 25% in 2016. For AMS interventions changing therapy based on cultures and sensitivities, whilst there was no discernible difference in the absolute number of interventions made per 1000 DDDs between 2013 and 2016, as a proportion of all AMS interventions this fell from 30% (2013) to 17% (2016). In contrast to previous years’ data, with the aid of the CDSS in 2016, a greater number of dose/drug optimisation (13.5% in 2016 vs 2.9% in 2013 and 5.3% in 2014) and escalation of antimicrobials (12.8% in 2016 vs 6.5% in 2013 and 5.3% in 2014) interventions were made. Despite this our hospital has still seen significant reduction in antimicrobial DDDs/1000 occupied bed days (OBD), from 283 pre-intervention to 231 post-intervention.

### Patient identification

The CDSS improved patient identification for review by the AMS team. Despite increased patient numbers post-CDSS rollout (from 1756 reviews in 2013 to 2664 reviews per 3 months in 2016) only 46 min on average was spent per day compiling a patient list for review, compared to 59 min pre-rollout. The system enabled identification of patients through a number of mechanisms. The user can set up alerts and reports to trigger when certain pre-determined conditions or parameters are met. Alerts were set by our hospital to identifying those patients with positive microbiology from invasive sites, these flag to the user when any new positive cultures are released by the laboratory or when cultures are updated with microbe identification or sensitivities. This allowed for patient review as soon as information was available, enabling a timely switch to appropriate antimicrobials. A separate alert triggers where ‘microbiology-antimicrobial’ mismatch of sensitivities has occurred, prompting an urgent review of the patient. Further reports automatically identify those receiving ‘restricted’ antimicrobials (carbapenems, oxazolidinones etc) or prolonged courses of non-restricted antimicrobials. The automated collation of this data allows for an increased selection of antimicrobials to be highlighted and has widened the net of patients to receive a stewardship review. Prior to intervention AMS was only provided on 60% of wards, neglecting paediatrics and obstetric wards. Post-implementation the only wards that are not captured are those with paper based prescribing, namely the intensive care unit (where a microbiologist attends daily ward rounds) and the neonatal intensive care unit. The introduction of CDSS facilitated 15 interventions/1000DDD, compared to pre-intervention baseline data (9.4/1000DDD in 2013 and 11.5/1000DDD in 2014).

### Mobile information

The introduction of CDSS has resulted in real time information of all antimicrobials, blood tests and microbiology being available on a tablet or laptop at the patient bedside. Prior to the implementation of CDSS, AMS interventions were predominately made from the microbiology office where full microbiology reports could be accessed. The CDSS intervention has enabled the AMS team to transition from an office based service into a patient facing, ward based service; reviewing notes, assessing patients, and documenting full infection specialist recommendations. The uptake of recommendations made by the AMS team to clinicians has increased significantly, previously 70% in 2013 and 71% in 2014, now 98% in 2016.

AMS team notes are written and stored on the CDSS to prompt future monitoring and interventions, streamline workflow, and aid handover between AMS team members.

### Real time data capture

Continuous input on the activity of the AMS team and the impact on patient care is automated through the CDSS. Reports can then be generated for any information collated by the CDSS, including for example on antimicrobial usage, instances of suboptimal escalation of antimicrobials, organism incidence, or antimicrobial resistance patterns at ward level. This enables continual audit of AMS practice allowing the team to identify areas of concern, including identifying particular specialities with high or inappropriate antimicrobial prescribing. In turn, over and above the daily operational AMS interventions, this was fed back to antimicrobial stewardship oversight committees enabling strategic AMS interventions around (i) policy change and (ii) specific clinical group re-education.

## Discussion

Our hospital has had an established AMS team for a number of years. With no change in team size the implementation of CDSS has resulted in increased activity, efficiency and effectiveness. Moreover CDSS implementation resulted in a shift in the focus of stewardship interventions on AMS ward rounds. The CDSS enabled a move away from simple targeting of interventions based upon dispensing records of ‘restricted’ antimicrobials. A mechanism that can be both time consuming and illustrative of a policing and restrictive AMS role. A 2017 Cochrane review demonstrated that restrictive AMS can lead to delays in treatment and negatively impact the relationship between the AMS team and the responsible clinical team [12]. Instead, use of the CDSS in this evaluation has widened AMS reviews to include escalation and lengthening antimicrobial courses and reformed AMS to be patient focused as opposed to antimicrobial focused. Feedback from all grades of doctors has been very positive on the emergence of the AMS team and the improvement to a less ‘policing’, more friendly and familiar consult team.

There are fiscal implications associated with all AMS practice, but understanding the whole healthcare economy of these can be circuitous. In England, recently set national quality and innovation (CQUIN) targets have significant financial implications for reductions in antimicrobial use [7]. This is in addition to the implications from AMR and inappropriate antimicrobial prescribing, both of which can worsen patient outcome [11, 15]. Specifically, these can be associated with increased length of stay, readmissions, and mortality, all of which also have financial implications for healthcare providers. The long term impact of this CDSS intervention, including on patient outcomes, length of stay, treatment failure and mortality, and the financial implications of CDSS adoption requires further evaluation. In particular, with the ever changing information technology landscape serving healthcare providers, the longevity of CDSS such as this must be considered with care.

In considering potential confounders of this evaluation, the release of the national antimicrobial stewardship CQUIN in 2016 coincides with the introduction of this CDSS and may have increased the number of interventions that focus on stopping antimicrobial therapy and changing antimicrobials based on MC&S and revised indication. Furthermore increasing healthcare professional and public awareness of AMR, and multiple national and international strategic level interventions may have also changed antimicrobial prescribing habits [16]. However these factors would not necessarily account for our reported data of CDSS-supported interventions optimising prescriptions towards escalation or increased course lengths in some cases. The evaluation was conducted retrospectively, therefore further limitations and cofounders are possible including changing patient demographics across the time periods, and others not identified, which may have affected results. A further limitation of the CDSS system is that it is not stand alone and requires expert users to interpret and appropriately utilise the presented information. This is particularly important where changes to laboratory culture identification (e.g. fine-resolution speciation following introduction of Matrix-Assisted Laser Desorption Ionisation-Time of Flight (MALDI-ToF) mass spectroscopy) and susceptibility testing change (e.g. increasing numbers of antimicrobials tested against each isolate). An expert user must ensure only clinically relevant results are followed through to the patient and have a sound understanding of antimicrobial resistance to prevent recommending sub-optimal antimicrobial therapy for non-significant isolates.

## Conclusion

Initial evaluation of the impact of CDSS on AMS at our hospital has demonstrated increased effectiveness and efficiency within the AMS team. The use of CDSS has improved patient case finding, AMS team productivity, and workflow auditing. More importantly, patient infection management has been optimised. It has contributed to the success of the healthcare provider in achieving nationally set remunerated AMS targets. Whilst confounding issues make analysis on length of stay, morbidity and mortality complex, future multicentre prospective work, in progress, will elucidate this area.

## Abbreviations

AMR: Antimicrobial resistance
AMS: Antimicrobial stewardship
CDSS: Computer decision support system
CQUIN: Commissioning for Quality and Innovation
DDD: Defined daily dose
HDU: High dependency unit
ICU: Intensive care unit
IV: Intravenous
MC&S: Microbiology culture and sensitivity
NHS: National Health Service
UK: United Kingdom
